# Hospitalized patients with COPD: analysis of prior treatment*

**DOI:** 10.1590/S1806-37132014000300005

**Published:** 2014

**Authors:** Irai Luis Giacomelli, Leila John Marques Steidle, Frederico Fernandes Moreira, Igor Varela Meyer, Ricardo Goetten Souza, Mariângela Pimentel Pincelli

**Affiliations:** Federal University of Santa Catarina, Florianópolis, Brazil; Federal University of Santa Catarina, Florianópolis, Brazil; Federal University of Santa Catarina, Florianópolis, Brazil.; Federal University of Santa Catarina, Florianópolis, Brazil.; Federal University of Santa Catarina, Florianópolis, Brazil.; Department of Clinical Medicine, Federal University of Santa Catarina, Florianópolis, Brazil

**Keywords:** Pulmonary disease, chronic obstructive, Pulmonary disease, chronic obstructive, Clinical protocols

## Abstract

**Objective:**

Although COPD is a prevalent disease, it is undertreated, and there are no available data regarding previous treatment of COPD in Brazil. This study aimed to determine the appropriateness of maintenance treatment in COPD patients prior to their hospitalization and to identify variables associated with inappropriate treatment.

**Methods:**

This was an observational, cross-sectional, analytical study involving 50 inpatients with COPD at two hospitals in the city of Florianópolis, Brazil. The patients completed a questionnaire on parameters related to the maintenance treatment of COPD. Non-pharmacological management and pharmacological treatment were assessed based on the recommendations made by the Global Initiative for Chronic Obstructive Lung Disease (GOLD) in 2011 and by the Brazilian National Ministry of Health in the chronic respiratory diseases section of its *Caderno de Atenção Básica* (CAB, Primary Care Guidebook).

**Results:**

In most of the patients, the COPD was classified as being severe or very severe. Regarding non-pharmacological management, 33% of the patients were smokers, only 32% had been advised to receive the flu vaccine, 28% had received pneumococcal vaccine, and only 6.5% of the patients in the B, C, and D categories received pulmonary rehabilitation. Regarding GOLD and CAB recommendations, pharmacological treatment was inappropriate in 50% and 74% of the patients, respectively. Based on GOLD recommendations, 38% were undertreated. A low level of education, low income, not receiving oxygen therapy, and not receiving the flu vaccine were associated with inappropriate treatment.

**Conclusions:**

The application of various non-pharmacological management recommendations was unsatisfactory. Regarding the GOLD recommendations, the high rate of inappropriate maintenance treatment was mainly due to undertreatment. In Brazil, even in severe COPD cases, optimizing treatment to achieve greater benefits continues to be a challenge.

## Introduction

A respiratory disease characterized by chronic airflow obstruction that is not fully reversible, with systemic manifestations, COPD is preventable and treatable, being associated with an abnormal inflammatory response (primarily to the inhalation of cigarette smoke, noxious particles, and toxic gases).^(^ ^1^ ^)^

The prevalence of COPD is high, as demonstrated by studies conducted in Brazil. Menezes et al. evaluated individuals over 40 years of age living in the metropolitan area of São Paulo, Brazil, and found that 15.8% had COPD.^(^ ^2^ ^)^ It has been estimated that there are over seven million adults with COPD in Brazil.^(^ ^2^ ^)^ The disease accounts for a large number of deaths, being the fifth leading cause of death in Brazil, and estimates indicate that COPD will have become the fourth leading cause of death in the country by the next decade.^(^ ^3^ ^)^

Underdiagnosis of COPD is common. In the study by Menezes et al., 88% of the patients with COPD had never undergone spirometry and therefore had an unconfirmed diagnosis of COPD.^(^ ^2^ ^)^ In a study evaluating patients treated at primary care clinics in the city of Aparecida de Goiânia, Brazil, 71% had never undergone spirometry and therefore had an unconfirmed diagnosis of COPD.^(^ ^4^ ^)^

Undertreatment of COPD is also common. Of the COPD patients in the metropolitan area of São Paulo in the last decade, only 2% reported having received a physician diagnosis of COPD, and only 18% reported that they were receiving treatment for the disease.^(^ ^2^ ^)^ The consequences of delayed, inappropriate treatment are disastrous and include increased exacerbations, loss of lung function, increased morbidity, and increased mortality.^(^ ^2^ ^,^ ^5^ ^)^ However, data on the maintenance treatment of COPD are scarce in Brazil.^(^ ^5^ ^)^

In the present study, we analyzed non-pharmacological management and the appropriateness of pharmacological maintenance treatment in COPD patients prior to their hospitalization at either of two referral hospitals for the treatment of respiratory diseases in the southern Brazilian state of Santa Catarina. We chose to evaluate inpatients because we assumed that they were more likely to present with COPD that is more severe and less likely to be underdiagnosed and undertreated (as a result of their having previously sought health care).^(^ ^6^ ^,^ ^7^ ^)^

In this context of unquestionable epidemiological relevance; that is, given that COPD is underdiagnosed and undertreated, the present study is warranted and was aimed at analyzing non-pharmacological treatment and the appropriateness of pharmacological maintenance treatment in COPD patients prior to their hospitalization. Non-pharmacological management and pharmacological treatment were assessed on the basis of the recommendations made by the Global Initiative for Chronic Obstructive Lung Disease (GOLD) in 2011^(^ ^1^ ^)^ and by the Brazilian National Ministry of Health in the chronic respiratory diseases section of its *Caderno de Atenção Básica* (CAB, Primary Care Guidebook).^(^ ^8^ ^)^ In addition, we sought to identify variables associated with inappropriate treatment in order to raise awareness to this problem and improve the treatment of COPD patients. 

## Methods

This was an observational, cross-sectional, analytical study conducted between December of 2012 and June of 2013 at the Polydoro Ernani de São Thiago University Hospital and the Nereu Ramos Hospital, both of which are located in the city of Florianópolis, Brazil. The aforementioned hospitals were chosen because they are referral centers for the treatment of respiratory diseases and are therefore expected to provide accurate diagnosis and appropriate treatment of COPD. 

The inclusion criteria were as follows: having been diagnosed with COPD on the basis of the 2011 GOLD criteria,^(1)^ the diagnosis being confirmed by spirometry; having been admitted to the pulmonology ward of either hospital; and agreeing to participate in the study by giving written informed consent. 

We used spirometric data obtained up to six months before admission, the most recent data being chosen. In the absence of earlier spirometric data, we used the spirometric data obtained after hospital discharge. 

The exclusion criteria were as follows: having been diagnosed with bronchial asthma, allergic rhinitis, or chronic lung diseases other than COPD; having any disease potentially affecting lung function; having never undergone spirometry; and not meeting the criteria for subsequent spirometry. 

During hospitalization, patients completed a structured questionnaire assessing the following: demographic data; socioeconomic data; non-pharmacological treatment-related factors, including smoking cessation, influenza vaccination, pneumococcal vaccination, home oxygen therapy, and pulmonary rehabilitation; and pharmacological treatment-related factors, including the use of short-acting β_2_ agonists, long-acting β_2_ agonists (LABAs), short-acting anticholinergics, long-acting anticholinergics, inhaled corticosteroids (ICs), and theophylline before admission. 

A total of 190 inpatients were initially selected. Of those, 53 had been diagnosed with COPD on the basis of spirometric data. Of those 53 patients, 3 were excluded because they also had asthma. The final sample consisted of 50 patients. 

In order to analyze the appropriateness of pharmacological treatment, we categorized patients, on the basis of the 2011 GOLD criteria, as follows: A (low risk and fewer symptoms); B (low risk and more symptoms); C (high risk and fewer symptoms); and D (high risk and more symptoms). Symptoms were assessed with the modified Medical Research Council scale.^(^ ^1^ ^)^ Exacerbations were defined as events requiring maintenance treatment modification.^(^ ^1^ ^)^ Patients were further classified as having mild COPD, moderate COPD, severe COPD, or very severe COPD on the basis of the CAB.^(^ ^8^ ^)^

We analyzed the appropriateness of treatment, as well as undertreatment and overtreatment, by comparing the treatment given with the treatment recommended in the 2011 GOLD guidelines. Undertreatment was defined as the complete absence of pharmacological treatment or the use of drug combinations other than those recommended for each category (A, B, C, or D). Overtreatment was defined as the unwarranted use of long-acting bronchodilators and ICs. On the basis of the 2011 GOLD parameters, the sum of the proportions of undertreatment and overtreatment resulted in the proportion of inappropriate treatment. A second analysis of the overall appropriateness of pharmacological treatment was performed on the basis of the CAB parameters. We opted to use the CAB guidelines and the 2011 GOLD guidelines because they represent the latest clinical protocols for COPD nationwide and worldwide, respectively. 

Statistical analysis was performed with the Statistical Package for the Social Sciences, version 15 (SPSS Inc., Chicago, IL, USA). Categorical variables were expressed as absolute numbers and proportions. We compared the distribution of categorical variables between the patients receiving appropriate treatment and those receiving inappropriate treatment on the basis of the 2011 GOLD guidelines using the chi-square test and, when necessary, Fisher's exact test. In addition, we used univariate analysis in order to identify factors potentially associated with inappropriate treatment in comparison with the treatment recommended in the 2011 GOLD guidelines, determining the crude OR and its confidence interval for that outcome. The level of significance was set at p < 0.05. 

The present study was approved by the Human Research Ethics Committee of the Polydoro Ernani de São Thiago University Hospital (Protocol no. 2425). All participants gave written informed consent.

## Results

We evaluated 50 inpatients with COPD (21 patients admitted to one of the aforementioned hospitals and 29 admitted to the other). Most of the patients were male, were over 65 years of age, were normal weight or overweight, had had up to 8 years of schooling, were retired, had a per capita family income of up to one time the national minimum wage, were former smokers, and had a smoking history of ≥ 20 pack-years. One third of the patients were active smokers. As can be seen in Table 1, most of the patients were classified as having disease that is more severe, 76% being classified into category C or D on the basis of the 2011 GOLD criteria and 64% being classified as having severe or very severe disease on the basis of the CAB criteria. Regarding knowledge of disease, 64% reported having emphysema, 64% reported having chronic bronchitis, and 40% reported having COPD.

**Table 1:** Demographic and socioeconomic data, as well as associated factors, together with the classification of the 50 COPD patients included in the study.

Variable		n	%
Gender	Male	33	66
Age, years	≤ 64	17	34
	≥ 65	33	66
Schooling, years	≤ 8	45	90
	≥ 9	5	10
Number of times the national minimum wage/number of people	≤ 1	31	62
	> 1	19	38
Occupational status	Not retired	17	34
	Retired	33	66
Body mass index, kg/m^2^	< 18.5	5	10
	18.5-24.9	22	44
	25.0-29.9	14	28
	> 30.0	9	18
Smoking status	Smoking	14	28
	Former smoker	33	66
	Passive smoker	3	06
Duration of dyspnea, years	< 10	19	38
	≥ 10	31	62
Physician-diagnosed emphysema, bronchitis, or COPD	Yes	47	94
2011 GOLD classification	A	2	04
	B	10	20
	C	5	10
	D	33	66
CAB classification (spirometry)	Mild	1	02
	Moderate	17	34
	Severe	26	52
	Very severe	6	12

GOLD : Global Initiative for Chronic Obstructive Lung Disease

CAB : *Caderno de Atenção Básica - doenças respiratórias crônicas* (Primary Care Guidebook, respiratory diseases section)

Regarding non-pharmacological maintenance treatment before admission, 14 patients were still smokers. Most (78%) reported that they had been advised by their physicians to quit smoking. Among those who had quit smoking, 95% had done so with no medications or cognitive behavioral therapy (Table 2).

**Table 2:** Non-pharmacological management prior to hospitalization in the 50 patients included in the study.

Variable	n/N	%
Having quit smoking	33/47	70
Having been advised by a physician to quit smoking	36/47	78
Having received influenza vaccination	40/50	80
Having been advised by a physician to receive influenza vaccination	16/50	32
Having received pneumococcal vaccination	14/50	28
Having been advised by a physician to receive pneumococcal vaccination	13/50	26
Being on home oxygen therapy	8/50	16
Undergoing pulmonary rehabilitation (GOLD categories B, C, and D)	3/48	6.5

GOLD : Global Initiative for Chronic Obstructive Lung Disease.

Regarding immunization, 88% of the patients had received influenza vaccination, 32% had been advised by their physicians to receive influenza vaccination, 28% had received pneumococcal vaccination, and a similar proportion of patients reported having been advised by their physicians to receive pneumococcal vaccination. Home oxygen therapy was used by 16% of the patients, and only half of those used it daily for ≥ 15 h. Only 6.5% of the patients in categories B, C, and D received pulmonary rehabilitation. 

Of the sample as a whole, 50% received inappropriate pharmacological treatment in comparison with that recommended in the 2011 GOLD guidelines. The proportions of inappropriate treatment in categories C and D were 60% and 33%, respectively. Of the sample as a whole, 74% received inappropriate treatment in comparison with that recommended in the CAB. Of the patients who were classified as having severe or very severe disease on the basis of the CAB guidelines, 69% and 50%, respectively, received inappropriate treatment (Table 3).

**Table 3:** Inappropriate pharmacological treatment for each COPD severity category in the 50 patients included in the study.

Classification	Category	Patients receiving inappropriate treatment	%
		(n/N)	
2011 GOLD	A	2/2	100
	B	9/10	90
	C	3/5	60
	D	11/33	33
	Total	25/50	50
CAB	Mild	1/1	100
	Moderate	15/17	88
	Severe	18/26	69
	Very severe	3/6	50
	Total	37/50	74

GOLD : Global Initiative for Chronic Obstructive Lung Disease

CAB : *Caderno de Atenção Básica - doenças respiratórias crônicas* (Primary Care Guidebook, respiratory diseases section)

Table 4 shows the use of different classes of maintenance medication among the different 2011 GOLD categories of patients. Of the sample as a whole, 14% received no pharmacological treatment at all. In addition, only 3 (6%) used theophylline, all of whom were category D patients.

**Table 4:** Pharmacological treatment regimens used for the different categories of COPD patients, classified on the basis of the 2011 Global Initiative for Chronic Obstructive Lung Disease guidelines.a

Categories	Patients (n)	SABA	SAA	SABA + SAA	LABA	LAA	LABA + LAA	IC	LABA + IC	LAA + IC	LABA + LAA + IC
A	2	0 (0)	0 (0)	0 (0)	0 (0)	0 (0)	0 (0)	0 (0)	1 (50)	0 (0)	0 (0)
B	10	2 (20)	0 (0)	3 (30)	1 (10)	0 (0)	0 (0)	0 (0)	1 (10)	0 (0)	3 (30)
C	5	1 (20)	0 (0)	0 (0)	0 (0)	0 (0)	1 (20)	0 (0)	1 (20)	0 (0)	1 (20)
D	33	16 (48)	6 (2)	7 (21)	1 (3)	2 (6)	1 (3)	2 (6)	4 (12)	2 (6)	13 (39)
Total	50	19 (38)	4 (2)	10 (20)	2 (4)	2 (4)	2 (4)	2 (4)	7 (14)	2 (4)	17 (34)

SABA : short-acting β_2_ agonist

SAA : short-acting anticholinergic

LABA : long-acting β_2_ agonist

LAA : long-acting anticholinergic

IC : inhaled corticosteroid

a Values expressed as n (%)

Of the sample as a whole, 38% were undertreated, 8% (patients in categories A and B) receiving no pharmacological treatment at all. For the remainder (30%), undertreatment was identified by the lack of use of short- or long-acting bronchodilators, as well as by a slight predominance of lack of IC use in patients in categories C and D. Overtreatment was found in 12% of the patients (in categories A and B), all of whom used ICs and 1 of whom used an LABA + IC combination (LABA + IC). 

In the univariate analysis, inappropriate pharmacological treatment (on the basis of the 2011 GOLD criteria) was significantly associated with up to 8 years of schooling, a per capita family income of up to one time the national minimum wage, and category A or B (i.e., less frequent exacerbations and FEV_1_ ≥ 50% of predicted),^(^ ^1^ ^)^ as can be seen in Table 5. Appropriate pharmacological treatment was significantly associated with the use of home oxygen therapy (OR = 9.33; 95% CI: 1.05-82.78) and influenza vaccination (OR = 5.41; 95% CI: 1.02-28.79), as well as with self-reported physician-diagnosed emphysema. 

**Table 5:** Variables associated with inappropriate treatment in comparison with the treatment recommended in the 2011 Global Initiative for Chronic Obstructive Lung Disease guidelines.

Variable		Treatmenta		p	OR	95% CI
		inappropriate	appropriate			
Per capita income, number of times the national minimum wage	≤ 1	14 (28)	6 (12)	0.021	4.03	1.20-13.53
	> 1	11 (22)	19 (38)			
Schooling, years	≤ 8	25 (50)	20 (40)	0.018	1.25	1.03-1.52
	> 8	0 (0)	5 (10)			
Physician-diagnosed emphysema	Yes	11 (22)	21 (42)	0.003	0.15	0.04-0.57
	No	14 (28)	4 (8)			
GOLD category	A or B	11 (22)	1 (2)	0.001	18.86	2.20-161.99
	C or D	14 (28)	24 (48)			

GOLD : Global Initiative for Chronic Obstructive Lung Disease.

## Discussion

The objective of the present study was to examine the appropriateness of maintenance treatment prior to hospitalization in patients with COPD that is more severe, a distinct strategy being used in order to select inpatients. Several aspects of the recommended non-pharmacological treatment were not followed. One third of the patients were smokers, a small proportion (28%) had received pneumococcal vaccination, and very few (6.5%) of the patients in category B, C, or D were undergoing pulmonary rehabilitation. Of the sample as a whole, 50% received inappropriate treatment in comparison with that recommended in the 2011 GOLD guidelines, undertreated patients having predominated. The proportion of patients receiving inappropriate treatment was even higher (i.e., 74%) when the CAB was used as a reference for treatment. Inappropriate treatment was found to be associated with a low level of education and low income. Appropriate treatment was found to be associated with physician-diagnosed emphysema, use of home oxygen therapy, and influenza vaccination.

The present study is the first of its kind in Brazil. In the international literature, there are few studies addressing the appropriateness of COPD treatment.^(^ ^2^ ^)^ In the present study, data collection was standardized, the same researchers having administered the questionnaire at both hospitals. 

In a study of the distribution of COPD patients by disease severity in Brazil,^(^ ^2^ ^)^ a household survey conducted in the city of São Paulo showed that the prevalence rates of mild, moderate, severe, and very severe COPD were 10.1%, 4.6%, 0.9%, and 0.2%, respectively. As expected (because of the strategy adopted), the prevalence rates in the present study were different from those in the general population: severe and very severe COPD (52% and 12%, respectively); and categories C and D (10% and 66%, respectively). Therefore, patients with COPD that is more severe predominated, the benefits of maintenance treatment being greater in such patients.^(^ ^5^ ^)^

The sociodemographic characteristics of the COPD patients in the present study were similar to those of those in the study by Menezes et al., who also found a higher prevalence of COPD among males who were over 65 years of age and had a significant smoking history.^(^ ^2^ ^)^

Various studies have shown that clinical protocols are not widely implemented. A study conducted in Brazil showed that 34% of general practitioners do not use clinical protocols for the management of COPD.^(^ ^9^ ^)^ Failure to follow the guidelines for the non-pharmacological maintenance treatment of COPD is consistent with the results of the present study; for example, 33% of the patients had not quit smoking, although smoking cessation has an impact on the rates of disease progression and mortality. Similar data are found in the international literature.^(^ ^2^ ^)^ In the present study, the proportion of patients who had been advised by their physicians to receive influenza and pneumococcal vaccination was low. Similarly, Menezes et al. found that only 30.6% of the COPD patients had received influenza vaccination, the proportions of vaccinated patients with severe and very severe COPD being 39% and 46%, respectively.^(^ ^2^ ^)^ A study evaluating COPD patients with respiratory failure admitted to the ICU also showed low vaccination rates, only 66.66% having received influenza vaccination and only 45.83% having received pneumococcal vaccination.^(^ ^10^ ^)^ Only 6.5% of the patients in the present study received pulmonary rehabilitation, which is recommended in the 2011 GOLD guidelines for patients in categories B, C, and D. Other studies have shown low rates of pulmonary rehabilitation. In one study, only 14% of the patients hospitalized for COPD exacerbation had received prior pulmonary rehabilitation.^(^ ^11^ ^)^

In the present study, it was impossible to evaluate the need for home oxygen therapy, given that we had no access to previous blood gas analyses. In addition, it is impossible to confirm the need for home oxygen therapy during clinical instability.^(^ ^1^ ^)^ Given the large proportion of patients with FEV_1_ < 50% of predicted (i.e., 64%) and the large proportion of category D patients (i.e., 66%), we can assume that the proportion of patients requiring home oxygen therapy was larger than that actually found (i.e., 16%). However, it is of note that half of the patients who were on home oxygen therapy did not receive it for as many hours per day as recommended, i.e. > 15 h.^(^ ^1^ ^)^

On the basis of the guidelines analyzed in the present study, pharmacological treatment was found to be inappropriate in 50-74% of the patients. Our data regarding inappropriate COPD treatment are consistent with those found in the literature. One group of authors evaluated patients who had moderate, severe, or very severe COPD and who had health insurance; the authors found that 43% of the patients had received medication prescriptions that were not in accordance with clinical protocols and that 51% had not obtained the medication.^(^ ^12^ ^)^

One reason for the difference between the two guidelines used in the present study in terms of the proportion of patients receiving inappropriate treatment is that the CAB does not recommend IC use for patients with moderate obstruction. However, in the present study, the patients with moderate obstruction had had a high number of exacerbations in the previous year and were therefore included in GOLD category C or D, for which IC use is recommended in the GOLD guidelines, meaning that a proportion of patients classified as having moderate COPD and into categories C and D used ICs. Using the 2011 GOLD guidelines as a reference for pharmacological treatment, we found that 38% of the patients were undertreated, undertreatment constituting the most common instance of inappropriate pharmacological treatment. In one third of those patients, treatment inappropriateness was due to the complete absence of pharmacological treatment. In the remaining patients, treatment inappropriateness was due to the lack of use of at least one of the classes of drugs recommended in the GOLD guidelines. 

It is known that COPD is underdiagnosed-88% of the COPD patients in the metropolitan area of São Paulo had an unconfirmed diagnosis of COPD-and undertreated.^(^ ^2^ ^)^ The present study included only patients with a diagnosis of COPD confirmed by spirometry; a confirmed diagnosis might be, in and of itself, a determining factor for better treatment. It is possible that the problem of undertreatment is even worse in patients with milder disease treated at less advanced centers and without a confirmed diagnosis. 

Overtreatment (i.e., inappropriate IC use) was found in 12% of the patients (GOLD categories A and B). This finding is relevant because overtreatment affords no clear benefits and increases the risk of hospitalization for pneumonia, as well as increasing the incidence of oral candidiasis, ecchymosis, and cataracts. ^(^ ^13^ ^-^ ^16^ ^)^ A recent systematic review showed that there is evidence for IC use only in patients with severe COPD and symptoms and in those with moderate or severe COPD, symptoms, and more than two COPD exacerbations per year.^(^ ^5^ ^)^ Incorrect prescription of LABA + IC is common among patients with moderate disease treated at primary or secondary health care facilities.^(^ ^17^ ^-^ ^20^ ^)^ Studies have shown that 45% of patients with moderate COPD receive ICs only, whereas 60% receive LABA + IC.^(^ ^21^ ^,^ ^22^ ^)^ In the present study, half of the patients in categories A and B used LABA + IC. One study showed that 50% of COPD patients admitted to the ICU for respiratory failure used systemic corticosteroids, meaning that at least 50% of the patients had received inappropriate non-pharmacological treatment.^(^ ^10^ ^)^ This reinforces the hypothesis that the use of clinical protocols is low, given that none of the major clinical protocols in Brazil include the use of systemic corticosteroids as a treatment option.^(^ ^1^ ^,^ ^8^ ^)^

Variables such as a low level of education and low income were significantly associated with a higher proportion of patients receiving inappropriate treatment. Given the context, this was expected. Inappropriate treatment was found to be significantly associated with being in category A or B. Given that the proportion of patients in those categories was low in the present study, it is impossible to determine whether treatment is most inappropriate in patients with disease that is less severe (in whom the number of hospitalizations is lower); if this is indeed the case, then health care is primarily focused on advanced disease. In contrast, appropriate treatment was significantly associated with home oxygen therapy and influenza vaccination. The external validity of the present study could have been higher if the number of participants had been larger. In addition, for the evaluation of maintenance treatment, different recruitment strategies can be used in order to increase the representation of all GOLD categories. 

In the present study, a physician diagnosis of emphysema was associated with an increased rate of patients receiving appropriate treatment. This might be due to the fact that patients give greater weight to the term emphysema and therefore seek treatment more often. 

The present study was conducted before the approval of the 2013 Brazilian National Ministry of Health clinical protocol and therapeutic guidelines for COPD and, therefore, before the proposal for providing COPD patients with specific medication.^(^ ^23^ ^)^ The present study can therefore serve as a historical control for future epidemiological studies and contribute to the reorganization of COPD care. 

On the basis of the evidence presented here, we conclude that the non- pharmacological management of COPD is currently unsatisfactory, as demonstrated by the significant proportion of active smokers, the low use of pneumococcal vaccination, and the lack of use of pulmonary rehabilitation. The same is true for the pharmacological treatment of COPD, the high rate of patients receiving inappropriate treatment being mainly due to undertreatment. Even in severe COPD cases, optimizing treatment to achieve greater benefits continues to be a challenge. Therefore, there is a need for further educating physicians and patients regarding COPD, as well as a need for more smoking cessation programs and pulmonary rehabilitation centers, together with a need for increasing the availability of medications for the treatment of COPD.

## References

[1] Global Initiative for Chronic Obstructive Lung Disease - GOLD (2011). Global Strategy for the Diagnosis, Management, and Prevention
of COPD - Revised 2011.

[2] Menezes AM, Perez-Padilla R, Jardim JR, Mui-o A, Lopez MV, Valdivia G (2005). Chronic obstructive pulmonary disease in five Latin
American cities (the PLATINO study): a prevalence study. Lancet.

[3] Sociedade Brasileira de Pneumologia e Tisiologia (2004). II Consenso Brasileiro de Doença Pulmonar Obstrutiva
Crônica (DPOC) - 2004. J Bras Pneumol.

[4] Queiroz MC, Moreira MA, Rabahi MF (2012). Underdiagnosis of COPD at primary health care clinics in
the city of Aparecida de Goiânia, Brazil. J Bras Pneumol.

[5] Menezes AM, Macedo SE, Noal RB, Fiterman JC, Cukier A, Chatikin JM (2011). Pharmacological treatment of COPD. J Bras Pneumol.

[6] Parker CM, Voduc N, Aaron SD, Webb KA, O'Donnell DE (2005). Physiological changes during symptom recovery form
moderate exacerbations of COPD. Eur Respir J..

[7] Barberà JA, Roca J, Ferrer A, Félez MA, Díaz O, Roger N (1997). Mechanisms of worsening gas exchange during acute
exacerbations of chronic obstructive pulmonary disease. Eur Respir J.

[8] Brasil. Ministério da Saúde. Secretaria de Políticas de Saúde.
Departamento de Atenção Básica. Doenças respiratórias crônicas (2010). Cadernos de atenção básica no 25.

[9] Aisanov Z, Bai C, Bauerle O, Colodenco FD, Feldman C, Hashimoto S (2012). Primary care physician perceptions on the diagnosis and
management of chronic obstructive pulmonary disease in diverse regions of the
world. Int J Chron Obstruct Pulmon Dis.

[10] Pincelli MP, Grumann AC, Fernandes C, Cavalheiro AG, Haussen DA, Maia IS (2011). Characteristics of COPD patients admitted to the ICU of
a referral hospital for respiratory diseases in Brazil. J Bras Pneumol.

[11] Donner CF, Virchow JC, Lusuardi M (2011). Pharmacoeconomics in COPD and inappropriateness of
diagnostics, management and treatment. Respir Med.

[12] Price L, Billups SJ, Rice MA, Hartsfield C (2007). Investigation of barriers to clinical practice
guideline-recommended pharmacotherapy in the treatment of COPD. Pharmacy Practice.

[13] Chung KF, Caramori G, Adcock IM (2009). Inhaled corticosteroids as combination therapy with
beta-adrenergic agonists in airways disease: present and future. Eur J Clin Pharmacol.

[14] Clark DJ, Grove A, Cargill RI, Lipworth BJ (1996). Comparative adrenal suppression with inhaled budesonide
and fluticasone propionate in adult asthmatic patients. Thorax.

[15] Calverley P, Pauwels R, Vestbo J, Jones P, Pride N, Gulsvik A (2003). Combined salmeterol and fluticasone in the treatment of
chronic obstructive pulmonary disease: a randomized controlled
trial. Lancet.

[16] Ernst P, Gonzalez AV, Brassard P, Suissa S (2007). Inhaled corticosteroid use in chronic obstructive
pulmonary disease and the risk of hospitalization for pneumonia. Am J Respir Crit Care Med.

[17] Glaab T, Banik N, Rutschmann OT, Wencker M (2006). National survey of guideline-compliant COPD management
among pneumologists and primary care physicians. COPD.

[18] Rutschmann OT, Janssens JP, Vermeulen B, Sarasin FP (2004). Knowledge of guidelines for the management of COPD: a
survey of primary care physicians. Respir Med.

[19] Jones RC, Dickson-Spillmann M, Mather MJ, Marks D, Shackell BS (2008). Accuracy of diagnostic registers and management of
chronic obstructive pulmonary disease: the Devon primary care
audit. Respir Res.

[20] Bourbeau J, Sebalt RJ, Day A, Bouchard J, Kaplan A, Hernandez P (2008). Practice patterns in the management of chronic
obstructive pulmonary disease in primary practice: the CAGE study. Can Respir J.

[21] Decramer M, Celli B, Kesten S, Lystig T, Mehra S, Tashkin DP (2009). Effect of tiotropium on outcomes in patients with
moderate chronic obstructive pulmonary disease (UPLIFT): a prespecified subgroup
analysis of a randomized controlled trial. Lancet.

[22] Tashkin DP, Celli B, Senn S, Burkhart D, Kesten S, Menjoge S (2008). A 4-year trial of tiotropium in chronic obstructive
pulmonary disease. N Engl J Med.

[23] Portal da Saúde (2012). Protocolo clínico e diretrizes terapêuticas doença pulmonar
obstrutiva crônica.

