# Supplementary material for: Hospitalized patients with COPD: analysis of prior treatment
Source: J Bras Pneumol. 2014 May-Jun;40(3):229–37. doi: 10.1590/S1806-37132014000300005 (PMC4109194; doi:10.1590/S1806-37132014000300005)
Supplement: Translation

# Translation: Pacientes portadores de DPOC hospitalizados: análise do tratamento prévio*

**Authors:** Irai Luis Giacomelli, Leila John Marques Steidle, Frederico Fernandes Moreira, Igor Varela Meyer, Ricardo Goetten Souza, Mariângela Pimentel Pincelli

**Affiliations:** Universidade Federal de Santa Catarina, Florianópolis (SC) Brasil; Universidade Federal de Santa Catarina, Florianópolis (SC) Brasil; Universidade Federal de Santa Catarina, Florianópolis (SC) Brasil; Universidade Federal de Santa Catarina, Florianópolis (SC) Brasil; Universidade Federal de Santa Catarina, Florianópolis (SC) Brasil; Departamento de Clínica Médica, Universidade Federal de Santa Catarina, Florianópolis (SC) Brasil

**Keywords:** Doença pulmonar obstrutiva crônica/terapia, Doença pulmonar obstrutiva crônica/prevenção & controle, Protocolos clínicos

## Abstract

**Objetivo:**

Embora a DPOC seja uma enfermidade prevalente, ela é subtratada, e dados sobre o tratamento prévio são desconhecidos em nosso meio. Buscou-se verificar a adequação às recentes diretrizes no que se refere ao tratamento de manutenção em pacientes com DPOC antes de sua hospitalização e identificar possíveis variáveis associadas à inadequação do tratamento.

**Métodos:**

Estudo transversal, observacional e analítico, que incluiu 50 portadores de DPOC, internados em dois hospitais na cidade de Florianópolis (SC). Aplicou-se um questionário sobre parâmetros relacionados ao tratamento de manutenção da DPOC. Avaliou-se o manejo não farmacológico e a adequação do tratamento farmacológico à terapia preconizada pelo *Global Initiative for Chronic Obstructive Lung Disease* (GOLD) 2011 e pelo *Caderno de Atenção Básica* (CAB) do Ministério da Saúde do Brasil sobre doenças respiratórias crônicas.

**Resultados:**

Na maioria dos pacientes, a DPOC foi classificada como grave ou muito grave. Em relação ao manejo não farmacológico, 33% eram tabagistas, apenas 32% foram orientados a receber vacinação anti-influenza, 28% receberam vacina anti-pneumocócica, e somente 6,5% dos pacientes nas categorias GOLD B, C e D realizaram reabilitação respiratória. O tratamento farmacológico foi inadequado em 50% e 74% da amostra, respectivamente, em relação às recomendações do GOLD e do CAB. Baseado nas recomendações do GOLD, 38% eram subtratados. Baixa escolaridade, baixa renda, não utilização de oxigenoterapia e ausência de vacinação anti-influenza associaram-se a inadequação do tratamento.

**Conclusões:**

Não foram seguidas satisfatoriamente várias recomendações do manejo não farmacológico. Segundo o GOLD, a elevada inadequação do tratamento de manutenção foi principalmente devida ao subtratamento. No Brasil, mesmo nos casos mais graves, a otimização do tratamento da DPOC para se obter benefícios mais evidentes continua a ser um desafio.

## Introdução

A DPOC é uma enfermidade respiratória prevenível e tratável, com manifestações sistêmicas, caracterizada pela presença de obstrução crônica ao fluxo aéreo, que não é totalmente reversível, e associada a uma resposta inflamatória anormal, principalmente à inalação de fumaça de cigarro, além da inalação de outras partículas e gases tóxicos.^(^ ^1^ ^)^

Essa enfermidade possui alta prevalência, constatada por estudos em nosso país. Menezes et al. avaliaram residentes na região metropolitana da cidade de São Paulo com mais de 40 anos de idade e encontrou 15,8% de indivíduos portadores de DPOC.^(^ ^2^ ^)^ Estima-se que haja mais de sete milhões de adultos brasileiros acometidos.^(^ ^2^ ^)^ A DPOC é responsável por um grande número de óbitos, sendo a quinta principal causa de mortalidade no país, com previsões para se tornar a quarta causa na próxima década.^(^ ^3^ ^)^

O subdiagnóstico dessa patologia é a regra. No estudo de Menezes et al. foi observado que 88% dos pacientes com DPOC não haviam realizado espirometria anteriormente e, portanto, não tinham confirmação diagnóstica.^(^ ^2^ ^)^ Isso se repetiu em 71% dos pacientes da rede de atenção básica de Aparecida de Goiânia avaliados em outro estudo.^(^ ^4^ ^)^

O subtratamento dessa doença também é frequente. Na região metropolitana de São Paulo, na última década, somente 2% da população acometida relatara haver recebido um diagnóstico médico da doença, e apenas 18% referira estar sendo tratada.^(^ ^2^ ^)^ A falta de tratamento adequado e precoce leva a consequências desastrosas para o paciente, como o aumento do número de exacerbações, a perda de função pulmonar e o aumento da morbidade e mortalidade.^(^ ^2^ ^,^ ^5^ ^)^ No entanto, em nosso meio, existem poucos dados sobre o tratamento de manutenção da DPOC.^(^ ^5^ ^)^

No presente estudo, foram selecionados pacientes portadores de DPOC, internados em dois hospitais de referência para o tratamento de doenças respiratórias em Santa Catarina, no sul do Brasil, de maneira a permitir a análise do manejo não farmacológico e a adequação do tratamento farmacológico de manutenção dedicada a esses pacientes anteriormente a sua internação. Escolheu-se avaliar pacientes internados, pois esses provavelmente apresentariam uma doença mais grave e com uma menor probabilidade de subdiagnóstico e subtratamento devido a um possível maior número de acessos prévios ao sistema de saúde.^(^ ^6^ ^,^ ^7^ ^)^

Nesse contexto de inquestionável relevância epidemiológica, devido ao subdiagnóstico e subtratamento dessa enfermidade, justifica-se o presente estudo, que visou analisar o tratamento não farmacológico e a adequação da terapêutica farmacológica de manutenção ofertada aos pacientes com DPOC, anterior a sua hospitalização, comparando ao recomendado pelas diretrizes da *Global Initiative for Chronic Obstructive Lung Disease* (GOLD) de 2011^(^ ^1^ ^)^ e do *Caderno de Atenção Básica* (CAB) do Ministério da Saúde do Brasil, que trata das doenças respiratórias crônicas.^(^ ^8^ ^)^ Além disso, o presente estudo teve como objetivo identificar possíveis variáveis relacionadas ao tratamento inadequado, de forma a poder alertar para esses fatores e aperfeiçoar o tratamento dispensado a esses pacientes.

## Métodos

Estudo transversal, observacional e analítico, realizado nas instituições Hospital Universitário Polydoro Ernani de São Thiago e Hospital Nereu Ramos, localizados em Florianópolis (SC), no período entre dezembro de 2012 e junho de 2013. Esses hospitais foram escolhidos por serem centros de referência para o tratamento de doenças respiratórias, nos quais se espera verificar uma melhor oportunidade de diagnóstico e de tratamento da DPOC.

Foram incluídos indivíduos com diagnóstico de DPOC confirmada por exame espirométrico, com base nos critérios do GOLD 2011,^(^ ^1^ ^)^ internados nos leitos da especialidade de pneumologia dos referidos hospitais, que estavam dispostos a participar da pesquisa e que assinaram o termo de consentimento livre e esclarecido.

A espirometria utilizada para a avaliação foi aquela apresentada pelo paciente, realizada até seis meses antes da internação, sendo escolhida aquela com data mais recente. Na ausência de espirometria prévia, foi utilizada a espirometria realizada após a alta da internação.

Os critérios de exclusão foram os seguintes: indivíduos com diagnóstico prévio de asma brônquica, rinite alérgica, doenças pulmonares crônicas outras que não DPOC, com enfermidades extrapulmonares capazes de interferir na função pulmonar, bem como aqueles que não possuíam espirometria prévia e/ou não preenchiam os requisitos para a posterior realização da espirometria.

Nos pacientes incluídos no estudo foi aplicado um questionário estruturado, durante a hospitalização, que avaliou dados demográficos, dados socioeconômicos, fatores relacionados ao tratamento não farmacológico -cessação do tabagismo, vacinação anti-influenza e antipneumocócica, oxigenoterapia domiciliar e reabilitação pulmonar - e fatores relacionados ao tratamento farmacológico - história de uso antes da internação de β2-agonista de curta ação, β2-agonista de longa ação (BALA), anticolinérgico de curta ação, anticolinérgico de longa ação, corticoide inalatório (CI) e teofilina. 

Foram identificados 190 pacientes hospitalizados durante o período do estudo; desses, 53 possuíam diagnóstico espirométrico de DPOC. Dos 53 pacientes, 3 foram excluídos por apresentar asma concomitantemente. A amostra final incluiu 50 pacientes.

Para analisar a adequação do tratamento farmacológico, os pacientes foram classificados, conforme o GOLD 2011, nas categorias A (baixo risco e poucos sintomas), B (baixo risco e mais sintomas), C (alto risco e poucos sintomas) e D (alto risco e mais sintomas). Os sintomas foram avaliados com a escala do *Medical Research Council* modificada.^(^ ^1^ ^)^ Exacerbações foram definidas como eventos nos quais foi necessária a modificação do tratamento de manutenção.^(^ ^1^ ^)^ Os pacientes foram ainda classificados como portadores de DPOC leve, moderada, grave ou muito grave, de acordo com o CAB.^(^ ^8^ ^)^

A avaliação da adequação do tratamento, bem como do subtratamento e do sobretratamento, foi realizada comparando-se o tratamento efetivamente utilizado e aquele preconizado pelo GOLD 2011. A ausência total de tratamento farmacológico ou o uso de combinações farmacológicas que não correspondessem às necessárias segundo o preconizado para as diferentes categorias (A, B, C e D) foram considerados como subtratamento. Considerou-se a utilização de broncodilatador de longa ação e de CI quando não havia indicação como tratamento inadequado (sobretratamento). Utilizando os parâmetros do GOLD 2011, a soma das proporções de subtratamento e de sobretratamento resultou na proporção de tratamento inadequado. Uma segunda análise do tratamento farmacológico quanto à adequação global foi realizada tomando os parâmetros do CAB como referência. Optou-se por utilizar tais diretrizes por representarem os protocolos clínicos para DPOC mais recentes no âmbito internacional e nacional, respectivamente. 

A análise estatística foi realizada utilizando-se a ferramenta *Statistical Package for the Social Sciences,* versão 15 (SPSS Inc., Chicago, IL, EUA). As variáveis categóricas foram descritas em termos de números absolutos e de proporções. A comparação da distribuição das variáveis categóricas entre os grupos de pacientes com tratamento adequado ou não segundo o GOLD 2011 foi analisada utilizando-se os testes do qui-quadrado e de Fisher, esse último quando necessário. Além disso, avaliaram-se os possíveis fatores associados à inadequação do tratamento, em relação ao proposto pelo GOLD 2011, através de análise univariada, determinando-se o OR bruto e seu intervalo de confiança em consideração a esse desfecho. Empregou-se um nível de significância p < 0,05. 

O presente estudo foi aprovado pelo Comitê de Ética em Pesquisa Humana do Hospital Universitário Polydoro Ernani de São Thiago, nº 2425. Os participantes concordaram em assinar o termo de consentimento livre e esclarecido.

## Resultados

Foram avaliados 50 pacientes hospitalizados (21 e 29 pacientes de cada instituição). A maioria dos pacientes era do sexo masculino, com mais de 65 anos de idade, eutrófico ou com sobrepeso, com até 8 anos de escolaridade, aposentado, com renda familiar per capita de até um salário-mínimo, ex-tabagista e com carga tabágica ≥ 20 maços-ano. Um terço dos pacientes era tabagista ativo. A maioria dos pacientes foi alocada nas classes de maior gravidade (GOLD 2011) C e D, em 76%, e grave e muito grave (CAB), em 64% (Tabela 1). Quanto ao conhecimento de sua doença, 64% dos pacientes relatavam ter enfisema, a mesma proporção informava ter bronquite crônica, e 40% informava ter DPOC.

**Tabela 1:** Dados demográficos, socioeconômicos, fatores associados e classificação dos 50 pacientes com DPOC incluídos no estudo.

Variáveis		n	%
Sexo	Masculino	33	66
Idade, anos	≤ 64	17	34
	≥ 65	33	66
Escolaridade, anos	≤ 8	45	90
	≥ 9	5	10
Número de salários-mínimos/número de pessoas	≤ 1	31	62
	> 1	19	38
Condição ocupacional	Não aposentado	17	34
	Aposentado	33	66
Índice de massa corpórea, kg/m^2^	< 18,5	5	10
	18,5-24,9	22	44
	25,0-29,9	14	28
	> 30,0	9	18
Condição tabágica	Tabagista	14	28
	Ex-tabagista	33	66
	Tabagista passivo	3	06
Duração de dispneia, anos	< 10	19	38
	≥ 10	31	62
Paciente já sabia ter enfisema, bronquite ou DPOC	Sim	47	94
Classificação GOLD 2011	A	2	04
	B	10	20
	C	5	10
	D	33	66
Classificação CAB (espirométrica)	Leve	1	02
	Moderado	17	34
	Grave	26	52
	Muito grave	6	12

GOLD : *Global Initiative for Chronic Obstructive Lung Disease*

CAB : *Caderno de Atenção Básica* - doenças respiratórias crônicas.

Em relação ao manejo não farmacológico de manutenção antes da internação, pôde-se observar que 14 pacientes ainda eram tabagistas. A maioria (78%) informava que havia sido orientada a parar de fumar por seu médico. Entre os que já haviam parado de fumar, 95% não utilizavam medicamentos ou terapia cognitivo comportamental (Tabela 2).

**Tabela 2:** Manejo não farmacológico anterior à internação nos 50 pacientes incluídos no estudo.

Variáveis	n/N	%
Cessou tabagismo	33/47	70
Cessação orientada por seu médico	36/47	78
Vacinação anti-influenza	40/50	80
Orientação médica para vacinação anti-influenza	16/50	32
Vacinação antipneumocócica	14/50	28
Orientação médica para vacinação antipneumocócica	13/50	26
Oxigenoterapia domiciliar	8/50	16
Reabilitação pulmonar (categorias B, C e D do GOLD)	3/48	6,5

GOLD : *Global Initiative for Chronic Obstructive Lung Disease*

Em relação à imunização, 88% dos pacientes receberam a vacina anti-influenza, 32% receberam orientação médica para essa vacinação, 28% dos pacientes receberam a vacina antipneumocócica, e uma proporção similar de pacientes referia ter sido orientada por seu médico a recebê-la. A oxigenoterapia domiciliar era utilizada por 16% dos pacientes, e apenas metade desses fazia uso diário por ≥ 15 h. Somente 6,5% dos pacientes dos grupos B, C e D realizavam reabilitação pulmonar.

O tratamento farmacológico observado em relação ao preconizado pelo GOLD 2011 era inadequado em 50% dos pacientes. As proporções de tratamento inadequado nas categorias C e D foram de 60% e 33%, respectivamente. Em relação às orientações do CAB, 74% dos pacientes apresentavam tratamento inadequado. Indivíduos graves e muito graves apresentaram 69% e 50% de tratamento inadequado com o CAB, respectivamente (Tabela 3).

**Tabela 3:** Inadequação do tratamento farmacológico em relação às classificações de DPOC nos 50 pacientes incluídos no estudo.

Classificação	Categoria	Pacientes com tratamento inadequado	%
		(n/N)	
GOLD 2011	A	2/2	100
	B	9/10	90
	C	3/5	60
	D	11/33	33
	Total	25/50	50
CAB	Leve	1/1	100
	Moderada	15/17	88
	Grave	18/26	69
	Muito grave	3/6	50
	Total	37/50	74

GOLD : *Global Initiative for Chronic Obstructive Lung Disease*

CAB : *Caderno de Atenção Básica* - doenças respiratórias crônicas

A Tabela 4 apresenta a utilização das diferentes classes de medicação de manutenção segundo as categorias GOLD 2011. Observou-se que 14% dos pacientes não recebiam qualquer tratamento farmacológico. Somente 3 pacientes (6%) faziam uso de teofilina, todos esses pertencentes à categoria D.

**Tabela 4:** Tratamento farmacológico utilizado nos pacientes classificados segundo as diretrizes do Global Initiative for Chronic Obstructive Lung Disease 2011.a

Categorias	Pacientes (n)	BACA	ACA	BACA + ACA	BALA	ALA	BALA + ALA	CI	BALA + CI	ALA + CI	BALA + ALA + CI
A	2	0 (0)	0 (0)	0 (0)	0 (0)	0 (0)	0 (0)	0 (0)	1 (50)	0 (0)	0 (0)
B	10	2 (20)	0 (0)	3 (30)	1 (10)	0 (0)	0 (0)	0 (0)	1 (10)	0 (0)	3 (30)
C	5	1 (20)	0 (0)	0 (0)	0 (0)	0 (0)	1 (20)	0 (0)	1 (20)	0 (0)	1 (20)
D	33	16 (48)	6 (2)	7 (21)	1 (3)	2 (6)	1 (3)	2 (6)	4 (12)	2 (6)	13 (39)
Total	50	19 (38)	4 (2)	10 (20)	2 (4)	2 (4)	2 (4)	2 (4)	7 (14)	2 (4)	17 (34)

BACA : β2-agonista de curta ação

ACA : anticolinérgico de curta ação

BALA : β2-agonista de longa ação

ALA : anticolinérgico de longa ação

CI : corticoide inalatório. aValores expressos em n (%).

A inadequação por subtratamento ocorreu em 38% dos pacientes de todas as categorias, e em 8% (categorias A e B) devido à ausência completa de tratamento farmacológico. Para o restante (30%), o subtratamento foi identificado pela não utilização de broncodilatadores de longa ação e/ou de curta ação, assim como um leve predomínio de falta de utilização de CI naqueles das categorias C e D. A inadequação por sobretratamento foi identificada em 12% dos pacientes (nas categorias A e B), todos pelo uso de CI e 1 pelo uso associado de BALA.

As características que se associaram à inadequação do tratamento farmacológico de acordo com o GOLD 2011 e que apresentaram significância estatística quando consideradas em análise univariada foram as seguintes: ter até 8 anos de escolaridade, ter renda familiar de até 1 salário mínimo por pessoa e ser alocado na categoria A ou B (que representam pacientes que têm menor frequência de exacerbações e com VEF_1_ ≥ 50% do previsto),^(^ ^1^ ^)^ conforme pode ser observado na Tabela 5. A utilização de oxigenoterapia domiciliar (OR = 9,33; IC95%: 1,05-82,78) e a vacinação anti-influenza (OR = 5,41; IC95%: 1,02-28,79) foram associadas de maneira significativa à adequação do tratamento farmacológico. Também a variável "médico informou diagnóstico de enfisema" (utilizando exatamente esse termo) associou-se à adequação do tratamento.

**Tabela 5:** Variáveis associadas com a inadequação do tratamento de acordo com o Global Initiative for Chronic Obstructive Lung Disease 2011.

Variáveis		Tratamento^a^		p	OR	IC95%
		não adequado	adequado			
Renda, salários mínimos per capita	≤ 1	14 (28)	6 (12)	0,021	4,03	1,20-13,53
	> 1	11 (22)	19 (38)			
Escolaridade, anos	≤ 8	25 (50)	20 (40)	0,018	1,25	1,03-1,52
	> 8	0 (0)	5 (10)			
Médico informou diagnóstico de enfisema	Sim	11 (22)	21 (42)	0,003	0,15	0,04-0,57
	Não	14 (28)	4 (8)			
Classificação GOLD	A ou B	11 (22)	1 (2)	0,001	18,86	2,20-161,99
	C ou D	14 (28)	24 (48)			

GOLD : *Global Initiative for Chronic Obstructive Lung Disease*.

## Discussão

O presente estudo propôs-se a analisar a adequação do tratamento de manutenção anterior à internação em portadores de DPOC de maior gravidade, utilizando uma estratégia diferenciada de captação durante a hospitalização. Várias recomendações do manejo não farmacológico preconizado não foram seguidas. Salientamos que um terço dos pacientes era tabagista, uma pequena proporção de pacientes recebeu vacinação anti-pneumocócica (28%) e, ainda, uma minoria dos pacientes das categorias B, C e D realizava reabilitação respiratória (6,5%). O tratamento farmacológico foi inadequado em 50% da amostra quando se considerou o GOLD 2011, com um predomínio de subtratamento. Quando o CAB foi a referência para o tratamento, a inadequação mostrou-se ainda maior (74%). Ressaltamos as seguintes associações que se relacionaram com a inadequação do tratamento: baixa escolaridade e baixa renda familiar. Vale destacar que as variáveis "diagnóstico de enfisema informado pelo médico", "utilização de oxigenoterapia domiciliar" e "vacinação anti-influenza" associaram-se com a adequação ao tratamento.

O presente estudo é original e inédito em nosso meio. Na literatura internacional existem poucos trabalhos publicados que abordam a adequação do tratamento da DPOC.^(^ ^2^ ^)^ No presente estudo, os mesmos pesquisadores aplicaram o questionário para a obtenção dos dados em ambas as instituições, o que confere homogeneidade à coleta de dados.

A distribuição dos pacientes com DPOC em relação à gravidade da doença foi estudada no Brasil através de um estudo,^(^ ^2^ ^)^ que mostrou através de um inquérito domiciliar realizado na cidade de São Paulo uma prevalência de DPOC de 10,1%, 4,6%, 0,9% e 0,2% de grau leve, moderado, grave e muito grave, respectivamente. No presente estudo, como se pretendia pela estratégia adotada, obtiveram-se taxas de frequência diferenciadas da população geral: DPOC grave e muito grave (52% e 12%, respectivamente); e categorias C e D (10% e 66%, respectivamente). Dessa forma, houve uma maior representação de pacientes nas classes de maior gravidade, nas quais o benefício do tratamento de manutenção é mais evidente.^(^ ^5^ ^)^

As características sociodemográficas encontradas no presente estudo são semelhantes às encontradas em pacientes portadores de DPOC no estudo de Menezes et al., que também observou uma maior prevalência da doença entre homens com mais de 65 anos e carga tabágica elevada.^(^ ^2^ ^)^

Diversos estudos reconhecem que os protocolos clínicos não são amplamente implementados: um estudo no Brasil demonstrou que 34% dos médicos generalistas não utilizam protocolos clínicos para o manejo da DPOC.^(^ ^9^ ^)^ A não observância das orientações preconizadas pelas diretrizes quanto ao manejo não farmacológico de manutenção pode encontrar paralelo nos resultados encontrados no presente estudo; por exemplo, 33% dos pacientes não cessaram o tabagismo, apesar de essa medida ter impacto sobre a taxa de progressão e de mortalidade da doença. Dados semelhantes são encontrados na literatura internacional.^(^ ^2^ ^)^ Notou-se uma baixa orientação médica em relação à vacinação anti-influenza e antipneumocócica. Menezes et al., de maneira semelhante, identificaram que somente 30,6% dos pacientes com DPOC receberam vacinação anti-influenza, sendo que nos grupos grave e muito grave essa proporção era de 39% e 46%, respectivamente.^(^ ^2^ ^)^ Através de um estudo que avaliou portadores de DPOC com falência respiratória admitidos em UTI, também foram demonstradas taxas insuficientes de vacinação (somente 66,66% e 45,83% receberam vacina anti-influenza e antipneumocócica, respectivamente).^(^ ^10^ ^)^ A reabilitação pulmonar, recomendada pelo GOLD 2011 aos pacientes pertencentes às categorias B, C e D, foi observada em somente 6,5% dos pacientes. Outros estudos também demonstram baixas taxas de reabilitação pulmonar. Em um estudo, apenas 14% dos pacientes internados por exacerbação da DPOC recebiam reabilitação pulmonar prévia.^(^ ^11^ ^)^

No presente estudo não se pôde avaliar a indicação de oxigenoterapia domiciliar, uma vez que não se tinha acesso às gasometrias prévias, além da impossibilidade de confirmar sua indicação durante a instabilidade clínica.^(^ ^1^ ^)^ Como havia uma grande proporção de pacientes com VEF_1_ < 50% do previsto (64%) e de pacientes na categoria D (66%), presume-se que haja um maior número de pacientes, em relação aos 16% encontrados, com indicação de uso de oxigenoterapia domiciliar. Entretanto, chama a atenção o fato de que metade dos pacientes que utilizava oxigenoterapia domiciliar não o fazia com a duração de horas diárias preconizadas, ou seja, por mais de 15 h diárias.^(^ ^1^ ^)^

De acordo com as diretrizes analisadas, vale destacar que o tratamento farmacológico mostrou-se inadequado em 50-74% dos pacientes. O presente estudo obteve dados similares de inadequação do tratamento aos da literatura. Um grupo de autores avaliou pacientes com DPOC de grau moderado, grave e muito grave que eram assistidos por um seguro de saúde e identificaram que 43% dos pacientes não possuíam medicação prescrita de acordo com os protocolos clínicos e que 51% não adquiriam a medicação.^(^ ^12^ ^)^

Um motivo para a diferença das proporções de inadequação do tratamento entre as duas diretrizes utilizadas seria o fato de que o CAB não orienta o uso de CI para pacientes com grau moderado de obstrução. Por outro lado, em nosso levantamento, esse grupo de pacientes apresentava um número elevado de exacerbações no último ano e, portanto, foram incluídos nas categorias C e D do GOLD, o qual, para essas categorias, indica o uso de CI; ou seja, uma parcela de pacientes classificados como com DPOC moderada e nas categorias C e D utilizavam CI. Em relação à adequação farmacológica baseada no GOLD 2011, o subtratamento representou o principal motivo de inadequação (38%). Em um terço desses, a inadequação foi devida à ausência completa de tratamento farmacológico. No restante, a inadequação deveu-se a não utilização de pelo menos uma das classes farmacológicas indicadas pelo GOLD.

Sabe-se que a DPOC é subdiagnosticada (88% dos pacientes da população metropolitana de São Paulo) e subtratada.^(^ ^2^ ^)^ No presente estudo, que incluiu apenas pacientes com DPOC confirmada por critério espirométrico, esse fato pode determinar uma maior probabilidade de que esses pacientes estejam sendo mais bem tratados somente pelo fato de que possuem um diagnóstico confirmado. Possivelmente na população com doença mais leve, tratada em centros de menor complexidade e sem acesso à confirmação diagnóstica, a situação de subtratamento deva ser pior. 

O sobretratamento ocorreu em 12% dos pacientes (categorias A e B do GOLD) pelo uso indevido de CI. Tal informação torna-se relevante pelo fato de esses pacientes estarem expostos, sem benefícios evidentes, a um maior número de hospitalizações por pneumonia, além da maior incidência de candidíase oral, equimose e catarata.^(^ ^13^ ^-^ ^16^ ^)^ Uma revisão sistemática recente mostra que a evidência para o uso de CI se estabelece somente para DPOC grave com a presença de sintomas e para DPOC moderada ou grave com presença de sintomas e mais de duas exacerbações por ano.^(^ ^5^ ^)^ Em pacientes com doença moderada, a prescrição incorreta de BALA + CI é comum na atenção primária e secundária.^(^ ^17^ ^-^ ^20^ ^)^ Estudos identificaram que 45% dos pacientes com gravidade moderada recebiam CI isoladamente, e 60% dos pacientes dessa mesma classe recebiam CI + BALA.^(^ ^21^ ^,^ ^22^ ^)^ No presente estudo, metade dos pacientes nas categorias A e B fazia uso de CI + BALA. Um estudo demonstrou que 50% dos pacientes com DPOC internados em uma UTI por falência respiratória faziam uso de corticoides sistêmicos, o que demonstra pelo menos uma taxa de 50% de inadequação do tratamento não farmacológico^(^ ^10^ ^)^ e reforça a hipótese de baixa utilização de protocolos clínicos, uma vez que os principais protocolos clínicos brasileiros não citam o uso de corticoides sistêmicos como uma opção de tratamento.^(^ ^1^ ^,^ ^8^ ^)^

Variáveis como baixa escolaridade e menor renda associaram-se significativamente com uma maior proporção de pacientes com tratamento inadequado. Esse fato pode ser interpretado como esperado nesse contexto. Observou-se que as categorias A e B apresentaram uma associação significativa para a inadequação do tratamento. Devido à baixa representação dessas categorias no presente estudo, podemos apenas observar esse comportamento sem poder confirmar se a maior inadequação do tratamento ocorre nas fases menos graves (as quais apresentam menos hospitalizações), o que poderia sinalizar uma atenção à saúde que atua predominantemente na doença avançada. Por outro lado, as variáveis relacionadas a oxigenoterapia domiciliar e vacinação anti-influenza se associaram, com significância estatística, à adequação do tratamento. Uma maior validade externa poderia ser obtida com um maior número de participantes. Diferentes estratégias de captação de participantes para permitir uma maior representação de todas as categorias GOLD poderiam também ser utilizadas para avaliar o tratamento de manutenção.

Adicionalmente, observou-se que quando os pacientes foram informados pelo médico do diagnóstico de enfisema, os pacientes apresentavam uma maior taxa de tratamento adequado, talvez por darem uma maior importância ao termo enfisema e, por isso, buscarem tratamento mais frequentemente. 

O presente estudo foi realizado antes da aprovação do protocolo clínico e das diretrizes terapêuticas para DPOC do Ministério da Saúde do Brasil de 2013 e, portanto, antes da proposta de fornecimento de medicação específica para os pacientes com essa doença.^(^ ^23^ ^)^ Dessa forma, o presente estudo pode servir como um indicador de controle histórico para futuros estudos epidemiológicos e colaborar para a reorganização do tratamento desses pacientes.

Diante das evidências descritas, conclui-se que o manejo não farmacológico da DPOC se apresenta insatisfatório, demonstrado pela proporção significativa de pacientes tabagistas ativos, pelo reduzido uso da vacinação anti-pneumocócica e pela falta de utilização de reabilitação pulmonar. O mesmo ocorre com o tratamento farmacológico, que possui elevada inadequação, sobretudo por subtratamento. Mesmo nos casos mais graves, a otimização do tratamento da DPOC para se obter benefícios mais evidentes continua a ser um desafio. Dessa forma, faz-se necessária uma educação ampliada dos médicos assistencialistas e da população acerca da doença, a implementação de mais programas de cessação do tabagismo e de um maior número de centros de reabilitação pulmonar, além do aumento da disponibilidade de medicações para o tratamento da DPOC.
